# Beyond the brain-computer interface: Decoding brain activity as a tool to understand neuronal mechanisms subtending cognition and behavior

**DOI:** 10.3389/fnins.2022.811736

**Published:** 2022-09-08

**Authors:** Célia Loriette, Julian L. Amengual, Suliann Ben Hamed

**Affiliations:** Institut des Sciences Cognitives Marc Jeannerod, CNRS UMR 5229, Université Claude Bernard Lyon 1, Bron, France

**Keywords:** brain decoding, brain–computer interfaces, machine learning, electrophysiology, fMRI, neurofeedback, cognition, attention

## Abstract

One of the major challenges in system neurosciences consists in developing techniques for estimating the cognitive information content in brain activity. This has an enormous potential in different domains spanning from clinical applications, cognitive enhancement to a better understanding of the neural bases of cognition. In this context, the inclusion of machine learning techniques to decode different aspects of human cognition and behavior and its use to develop brain–computer interfaces for applications in neuroprosthetics has supported a genuine revolution in the field. However, while these approaches have been shown quite successful for the study of the motor and sensory functions, success is still far from being reached when it comes to covert cognitive functions such as attention, motivation and decision making. While improvement in this field of BCIs is growing fast, a new research focus has emerged from the development of strategies for decoding neural activity. In this review, we aim at exploring how the advanced in decoding of brain activity is becoming a major neuroscience tool moving forward our understanding of brain functions, providing a robust theoretical framework to test predictions on the relationship between brain activity and cognition and behavior.

## Introduction

One of the major challenges of system neurosciences is to understand how brain functions subtends cognition and behavior. This knowledge is essential not only for a better description of how the brain works, but also to develop strategies to boost cognition and to ameliorate, or even restore, cognitive functions affected by neurological diseases. During the last decades, a humongous technical, theoretical, and clinical effort has been invested in exploring and analyzing the activity of the brain. One important advance in this context that is gaining a huge momentum in the field is the inclusion of machine learning to the analysis of brain activity (Abraham et al., 2014; Glaser et al., 2020; Iturrate et al., 2020). Machine learning is a branch of artificial intelligence which consists in the development of algorithms that imitate the way humans learn from data. The main motivation of the application of machine learning in neuroscience is the development of brain–computer interface technologies (BCI), which comes with the idea that computers might be able to mimic some of the brain’s most basic cognitive capacities (Johnson, 2000). In other words, computers might reproduce the type of computations (or cognitive operations) performed by the brain. This does not imply biomimetism. In particular, it does not imply that machine learning imitates how humans learn. For instance, these techniques might be able to learn predicting a specific output (specific movement or a physical attribute of an object) from given states of a system (the activity of a region of the brain) (Glaser et al., 2020) or identifying which activity patterns are more alike to a certain behavioral counterpart (Richards et al., 2019). To achieve these goals, machine learning refers to a large list of methods spanning from supervised linear regression algorithms to other more sophisticated and complex learning tools such as deep learning neural networks.

A seminal application of machine learning in the field of neuroscience consists in the classification of brain activity patterns to predict observable outputs such as motor behavior (e.g., arm or leg movements) and visual inputs (physical attributes of stimulus) (Zafar et al., 2015; Branco et al., 2017; Contini et al., 2017; Tam et al., 2019; Kashefi and Daliri, 2021; Nazari et al., 2021) achieved in real-time, which has allowed the development of brain–computer interface technologies (BCI). Nowadays, such technologies have allowed, for instance, the real-time reconstruction of an image seen by a subject from the analysis of the concurrent visual responses recorded in the subject’s occipital cortex (Shen et al., 2019; Huang et al., 2020) or to control a robotized arm by using exclusively the neural activity recorded over the motor cortex of the subject. This has led to the development of the research field of neuroprosthetics, with a myriad of potential applications to restore lost motor and sensory brain functions (Hochberg et al., 2006, 2012; Adewole et al., 2016; Bouton et al., 2016; Lebedev and Nicolelis, 2017). However, and while most of the research in neuroprosthetics fall into the use of signals associated to observable outputs such as those commented above, a whole range of cognitive functions remain unexploited in this context, such as attention, memory, visual imagery and motivation, and predicting these covert cognitive functions from brain activity remains challenging (Astrand et al., 2014b).

In addition to the development of BCI interfaces to provide tools for neuroprosthetics, a new perspective on machine learning in neuroscience is emerging, which consist on its use to genuinely model different aspects of brain processes and, thus, increase our understanding of brain functions (Hebart and Baker, 2018). In this context, relevant advances have been achieved in understanding covert spatial attention mechanisms, which corresponds to the ability of a subject to select relevant sensory information while ignoring other inputs or stimulations, independently of eye position. Visual covert attention is known to rely on a well described brain network involving the prefrontal cortex and specifically the frontal eye fields (FEF), the intra-parietal sulcus (IPS) and striate and extra-striate visual areas (Shulman et al., 1999; Corbetta and Shulman, 2002; Bisley, 2011; Bogadhi et al., 2018). Behavioral evidence has suggested that attention rhythmically samples space (Buschman and Miller, 2007; VanRullen et al., 2007; Fiebelkorn et al., 2013, 2018; Dugué et al., 2015, 2016; VanRullen, 2016, 2018; Gaillard and Ben Hamed, 2020; Gaillard et al., 2020). The use of machine learning tools to predict the position of the attentional spotlight have revealed that the position of the spatial attention oscillates, at the same frequency as the behavioral performance rhythmic fluctuations. Therefore, machine learning tools have been useful to describe the neural bases accounting for the behavioral attentional sampling fluctuations (Gaillard et al., 2020).

The aim of this review is to explore how advanced machine learning methods, beyond their applicability in neuroprosthetics, can be used as a powerful tool to better characterize brain functions, with a specific focus on covert functions. First, we will introduce the concept of *decoding* of brain activity and its application into multiple cognitive brain functions, and we will discuss the different methods of brain decoding. Second, we will provide an overview of the specific methodology that machine learning offers to neuroscientists to describe the relationship between brain activity and cognitive brain functions. Last, we will show how we can use this knowledge to develop accurate cognitive brain–computer interface tools based on neurofeedback and learning.

## Decoding brain information

### Input cortical signals

The aim of machine learning in neuroscience is to extract reliable information associated to a specific cognitive function subtended by brain activity. These computational methods are based on neural decoding, which consists in the ability of an algorithm to predict or reconstruct the information that has been encoded and represented in the activity of a specific brain region or network. In this section we will discuss the different methods for extracting brain activity and the different advantages and disadvantages related to decoding of information.

**FIGURE 1:**
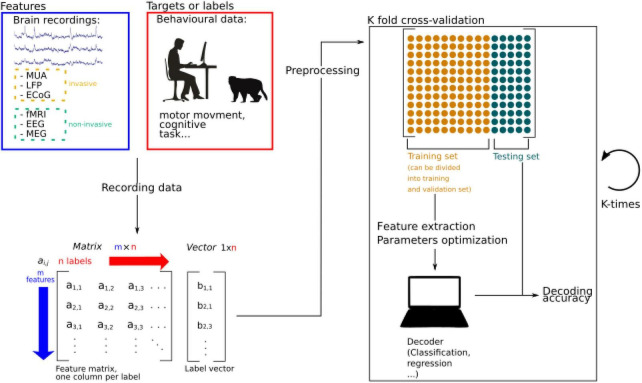
Schematic representation of machine learning procedures applied to brain decoding. Brain activity is recorded while participants are performing a specific behavioral task. The recorded data is stored as a matrix and data is pre-processed to extract relevant information and increase the signal to noise ratio. Extracted information is called features. Then the data is split into two sets, a training, and a testing set. The training set can further be divided into a training set and a validation set, used to perform feature extraction and parameters optimizations. If the algorithm is supervised, features and labels are fed to the decoder. In unsupervised learning, only the features are used to train the algorithm. The performance of the decoder is then estimated by testing its accuracy thanks to the remaining testing set. Splitting the data into training and testing sets can be done by splitting the data into *N* sets (*N* is a number to be defined) allowing to generalize the performance of the algorithm by calculating an average decoding accuracy.

Broadly speaking, brain activity can be recorded either using invasive or non-invasive methods (Figure 1). Invasive electrophysiological recordings allow to record single-unit (SUA) or multi-unit activity (MUA), reflecting the activity of a few neurons, and corresponding to discrete action potentials, which can be seen as bits of neuronal information. They also allow to record the activity of a larger neuronal population, including their synaptic inputs and outputs, called local field potentials (LFP). To note, the size of the corresponding neuronal population reflected in the LFP depends on the physical impedance of the implanted electrodes (the lower the impedance, the larger the neuronal population that the LFP represents). These signals are characterized both by a very high temporal resolution and signal-to-noise ratio allowing a single trial level decoding. Indeed, this signal is recorded using electrodes that are purposefully implanted in cortical regions that are specifically involved in processing the function of interest. However, these methods also have a quite low spatial resolution as they sample only a few cortical sites, even when dense multi-unit recordings are performed, and therefore they are restricted to specific brain sources. Mostly used in non-human primate experiments, this type of invasive approach has led to massive advances in the field of motor neuroprosthetics. Previous studies have shown in non-human primates the possibility to drive robotic arms (Velliste et al., 2008) or virtual effectors (Golub et al., 2014) using a direct control over the activity of their motor cortex. These approaches have also been applied to covert cognitive functions, that is to say, to the decoding of the content of the cognitive processes rather than the observable associated behavior (Astrand et al., 2014a). In this context, important advances have been obtained in tracking spatial attention (irrespective of eye position) with both a high spatial (in the order of 0.5° visual degree) (Astrand et al., 2016; De Sousa et al., 2021) and a high temporal resolution (in the order of 50 ms) (Gaillard et al., 2020). Invasive experimental set ups have also been developed in human studies, whether using electrocorticography (ECoG), or intracranial electroencephalography (iEEG), but strictly restricted to specific clinical demands. Some studies have included tetraplegic patients applying decoding methods to their cortical activity in order to restore their motor functions (Hochberg et al., 2012; Bouton et al., 2016; Ajiboye et al., 2017). However, and in spite of the refined and precise information that they provide, the application of invasive methods of decoding brain activity in humans remains rare as they involve an invasive surgery and they come with strong ethical limitations (Nicolas-Alonso and Gomez-Gil, 2012).

Fortunately, brain activity can also be recorded using non-invasive techniques such as scalp electroencephalography (EEG) which provides recording activity at a very high temporal but low spatial resolution, and a very low signal-to-noise ratio; magnetoencephalography (MEG), which has a very good temporal and cortical spatial resolution, in spite of a variable signal-to-noise ratio; and functional magnetic resonance imaging (fMRI), which allows to record whole-brain activity with a high spatial resolution, but is limited temporally by the time of acquisition of the different brain slices and the huge delay (in the order of few seconds) of the hemodynamic response (BOLD signal) relative to the actual neuronal response, as well as by a relatively low signal-to-noise ratio (Nicolas-Alonso and Gomez-Gil, 2012). Another example of non-invasive methods of brain recording used for decoding information is the use of functional-near infrared spectroscopy (fNIRS), which is characterized by a higher spatial resolution than the non-invasive electrophysiological recordings (EEG/MEG) and much better temporal resolution than the fMRI, as well as by a higher portability than the abovementioned non-invasive methods (Wilcox and Biondi, 2015).

Despite these limitations, non-invasive methods of brain activity recording have shown quite successful decoding capacities. For instance, the lateralization of the locus of spatial attention has been decoded in humans using event related brain potentials (ERPs) extracted from scalp EEG recordings in humans in response to visual presentations (Trachel et al., 2015; Thiery et al., 2016). In fMRI, covert visuospatial attention can be decoded with a high level of accuracy when four positions are encoded (80% of accuracy) (Andersson et al., 2012) and 40% accuracy when eight positions are encoded (Loriette et al., 2021). Machine learning has also been applied to train predictive speech models using fNIRS, obtaining a 75% of accuracy in classifying long speech segments from brain activity (Liu and Ayaz, 2018).

Up-to-know, the current decoding of information capacities from non-invasive brain signal shows lower accuracies when compared with those obtained with invasive techniques. However, this limitation is overcome by the fact that they are readily accessible, easy to manipulate and “discomfort-free” by the user. Therefore, an intense effort is invested in the field to improve the decoding capacities using such methods to develop more successful brain–machine interface and other therapeutic applications.

### Methodology of brain decoding

Machine learning methods rely on the development of algorithms to map recorded brain activity onto encoded information. These algorithms can be classified into *supervised* or *unsupervised* learning algorithms. Supervised algorithms learn the mapping between an input to an output by using input-output pairs from a set of *training* examples. From such learning process, an inferred function is provided that can be used to map new examples onto the different possible outputs (Figure 1). For instance, a function can be trained to identify whether a movement will be performed using the left or the right upper limb by providing multiple datasets of recorded brain activity from the left and right motor cortex concomitant with the movement of both arms. Once the algorithm has learned this association, it will be able to predict which arm is being moved by merely using novel brain recordings from both hemispheres. In contrast, *unsupervised* learning involves feeding a model with brain activity without giving any explicit information about the corresponding output, and therefore letting the algorithm estimate the number of possible outputs from a classification of the activity of the data based on its multidimensional statistical structure. New observations are then associated with these statistically defined classes. There exists a very large range of supervised and unsupervised classification algorithms which we will not discuss here as this would require an independent review in itself.

Some considerations must be considered when selecting which learning algorithm is more suitable to use. Unsupervised learning algorithms are quite sensitive to the size of the data (dimensionality and number of trials). It is well known that complex models such as deep neural network (networks with multiple layers) based learning algorithms require large data sets to obtain a good model estimation. In the absence of large enough data sets, these models risk overfitting, which consists in the over-learning of the training data structure, producing a low generalization of the prediction capacities when novel data sets are used. The risk of overfitting is precisely the reason why deep neural networks are often restricted to overt visual or motor data which can be collected in large amounts in a short time. Other lower complexity models such as support vector machine (SVM), linear discriminant analysis (LDA) or regression trees are applied to the decoding of covert cognitive functions, as these require more demanding and longer behavioral tasks to collect training data (Lemm et al., 2011; Abraham et al., 2014; Taghizadeh-Sarabi et al., 2015).

When considering supervised learning algorithms, specific good practices should be followed regarding training and testing procedures. The aim of these algorithms is to estimate the weights of a more or less complex function which minimizes the prediction error of the training set (i.e., a model which minimizes the error between the actual outputs of the training set and the outputs predicted by the model from the brain activity associated with the actual outputs of the training set). However, the prediction capabilities of the model (the decoding accuracy) are generally measured by using a different set of novel inputs (testing set) and evaluating the prediction error in these new examples. The standard methodology used to achieve a reliable decoding accuracy is the cross-validation, which consist in dividing or splitting the data set iteratively in two training and testing subsets. The decoding accuracy in these dataset will be defined as the average of the accuracies obtained in each iteration from different training and testing sets (Glaser et al., 2020) (Figure 1).

Last, most of the models are characterized by tuning parameters that can be changed to refine the model. As is the case for training, these parameters cannot be optimized on the whole data set as this will inflate the accuracy scores. Most often, the dataset is divided into three sets, one set for parameter optimization (called the validation set), one set for training data on the optimized model and one set for testing and estimating an unbiased decoding accuracy. As described from training/testing, cross-validation approaches can also be applied to model parameter optimization and to select the main features of the data prior to model training (Glaser et al., 2020).

## Exploring cognitive brain function using decoding methods

### Decoding accuracy as a window into cognitive brain processes

Decoding information related with covert cognitive functions such as attention, intention, and decisions are still in its early days. Indeed, decoding covert information often leads to lower decoding accuracies as compared to the decoding of sensory or motor functions, despite consistent effort invested to improve this decoding. This stems from multiple reasons. The first reason pertains to the fact that cognitive information (say spatial attention) is implemented in associative cortices, mixed with multiple other sources of cognitive information (e.g., working memory, temporal expectation, planning etc.). Such multiplexing of cognitive information has been theorized as a strategy to enhance the coding dimensionality of primate brains (Rigotti et al., 2013). In the absence of an appropriate pre-processing of the neuronal data, this results in a low signal-to-noise (SNR) when trying to extract a given dimension in isolation. In other words, signal corresponding to the specific cognitive process of interest might be too low compared to other sources of information contained in that same signal. Such effect has been well described in specific brain regions, such as the prefrontal cortex, in which different sources of information are simultaneously encoded, a property of neuronal populations known as mixed selectivity (Rigotti et al., 2013). With appropriate pre-processing, such as dimensionality reduction or de-mixed dimensionality reduction approaches (Kobak et al., 2016), SNR is enhanced, as it becomes possible to assign overall signal variance to the process of interest as well as to the cognitive processes of non-interest. Applying such dimensionality reduction approaches to MUA recordings form the FEF allows to better decode the spatial orientation of attention irrespective of whether the subjects are engaged in the task of not, thus dissociating between attention orientation and task engagement (Amengual and Ben Hamed, 2021; Amengual et al., 2022).

**FIGURE 2:**
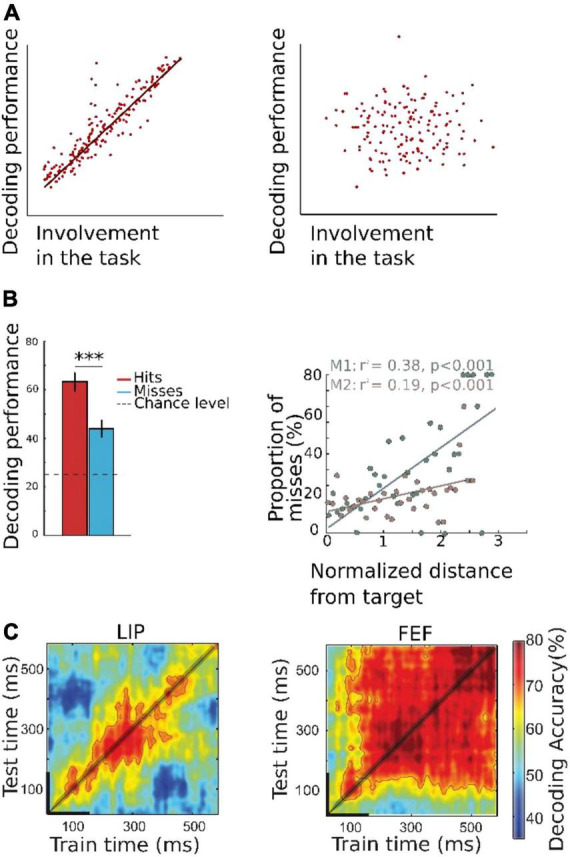
**(A)** Schematic representation of theoretical relationship between decoded information and behavioral proxy of decoded internal brain state. Left: Decoded information is highly correlated with the underlying cognitive brain state, and the behavioral proxy of this decoded brain information (hit, misses, reaction time) is strongly correlated with the decoded information. Right: If the decoder is not a good estimator of covert brain information, there is no correlation between decoded information and the behavioral proxy to the decoded cognitive function. Please note that this type of approach is more robust when the output of the decoder is continuous and non-categorial. **(B)** Decoded spatial locus of attention correlates with behavioral performance. Decoding was performed using MUA activity recorded from two rhesus monkeys, while the animals played a cued target detection task. On each trial, a cue was presented to the monkeys who were trained to keep their gaze at the center of the screen while orienting their covert attention to the cued location (one amongst four possible locations), to detect. The trained algorithm aimed at decoding the quadrant to which attention was oriented (left) or the actual (*x-*,*y-*) location of the attentional spotlight (right). Left: Decoding accuracy as a function of behavioral performance. Decoding accuracy is higher for correct detection trials than for miss trials, in which the monkeys did not properly identify the target, indicating that the decoding algorithm is able to capture internal attentional orientation information. Right: Proportion of misses as function of distance between the target and the decoded position of attention in *X*-*Y*- coordinates. Farther is the decoded position from the target at time of presentation, higher is the probability of missing the target (adapted from Astrand et al., 2016). **(C)** Temporal generalization of two algorithms trained with MUA activity from two different brain regions known to have a role in covert attention: lateral intraparietal area LIP (left) and frontal eye field FEF (right). The algorithm is trained on successive times intervals and for each training interval, tested on all available time intervals (cross-temporal decoding). This highlights two distinct neuronal population coding schemas: a dynamic coding in LIP whereby the neuronal code for attention orientation at a given time interval does not generalize to other time intervals, and a static coding in FEF, whereby the code identified at a given time generalizes at all times following cue presentation (Astrand et al., 2015). ***p < 0.001.

The second reason why decoding cognitive information does not reach high accuracies is due to the fact that, in contrast with motor or sensory responses which can be precisely timed relative to movement initiation or sensory stimulation onset, cognitive information is generated endogenously by the subjects, such that onset time can only be approximated from external events. For example, when producing an arm movement in response to a visual cue, subjects will produce a range of motor response from fast to slow. When instructed to orient their spatial attention following a visual cue, subjects will also do so more or less rapidly. However, there is no objective way of quantifying this precisely. Likewise, while an awkwardly organized arm movement can directly be observed, a sluggish attentional orientation can only be inferred from success in the ongoing task. This thus results in uncontrolled for sources of noise. A good example to illustrate this is the strategy that our research group has implemented to decode spatial attention orienting. Astrand et al. (2016) decode the (*x−*,*y−*) position of attention by training an algorithm on correct trials only, under the very strong assumption that on such trials, attention is precisely oriented at the cued location. The decoding output on the test trials makes it clear that this assumption is false not only on error trials (where one expects attention to be miss allocated) but also on correct trials, such that attention can be either close or far to the cued location, although on average, attention is positioned on the cued location (Astrand et al., 2016, 2020). These findings have been confirmed in humans using fMRI recordings (Loriette et al., 2021). Indeed, when predicting the spatial orienting of attention from BOLD activity in the striate and extrastriate cortex, while maximum decoding accuracy is achieved for the spatial location that the subjects are requested to attend to, on a significant proportion of the trials, attention is actually localized around the instructed location, thus indicating that attention is not always anchored at the cued location. Importantly, decoding output still strongly accounts for behavioral performance, such that the closer the decoded attention to the cued location at time of target presentation, the lower the probability that the monkeys produce a miss (so the higher the probability of a correct detection, Figures 2A,B) (Astrand et al., 2016, 2020). Based on recent advances in machine learning (Lemm et al., 2011; Abraham et al., 2014; Savage, 2019; Glaser et al., 2020; Iturrate et al., 2020), we thus reasoned that not all correct trials shared the same degree of attention-related information and we trained a second decoder selecting only those correct trials that predicted attention closest to the cued location from the initial decoding step (De Sousa et al., 2021). This remarkably enhanced the degree of correlation between the distance between decoded attention and the cued location on the one hand and success of the subjects in the task on the other hand. This was true whether attention was decoded from MUAs, or from LFPs (Esghaei and Daliri, 2014; Seif and Daliri, 2015; De Sousa et al., 2021), suggesting that such achievements could also be expected from ECoG, EEG or MEG signals. Indeed, pioneer studies have obtained remarkable accuracy thresholds in decoding attention using non-invasive recordings such as EEG (Treder et al., 2011; O’Sullivan et al., 2015) and MEG (Battistoni et al., 2018; Desantis et al., 2020). The above studies rely on attentional tasks such that decoding attention accuracy can be confronted with the resultant behavioral attentional bias. Quite interestingly, decoding of attention can be achieved even when attention does not bias behavior, i.e., when subjects are engaged in cognitive tasks that do not explicitly monitor the attentional function (Westendorff et al., 2016), suggesting that decoding attention can be performed outside of a controlled laboratory setup.

A third reason why decoding of cognitive variables might be suboptimal is our proper understanding of how this function interacts with other cognitive functions or a proper understanding of its spatial and temporal properties. For example, it has been very intriguing to us to observe that error trials could still be produced even when spatial attention was decoded close to the cued location (Astrand et al., 2016, 2020; De Sousa et al., 2021). De-mixed dimensionality reduction approaches (Kobak et al., 2016), allowed us to demonstrate that spatial attention orientation organizes in the prefrontal cortex distinctly from engagement in the task, such that target miss-detections could arise from both an inappropriate allocation of attention or an inappropriate engagement in the task (Amengual and Ben Hamed, 2021; Amengual et al., 2022). This biologically-inferred decoding schema further enhances our accuracy at tracking the actual spatial spotlight of attention and better account for its contribution to overt behavior.

Another example is the recent understanding we gained on the dynamical structure of decoded spatial attention exploration and exploitation (Gaillard and Ben Hamed, 2020; Gaillard et al., 2020), corroborating a large field of behavioral (VanRullen et al., 2007; Landau and Fries, 2012; Fiebelkorn et al., 2013, 2018; Dugué et al., 2015, 2016; VanRullen, 2016, 2018) and electrophysiological (Fiebelkorn et al., 2018) body of research. Specifically, we show that rather than being stable at a given location in space, the attentional spotlight explores space rhythmically, at 8 Hz, alternating between epochs of exploration away from the task relevant locations, and epochs of exploitation, at task relevant locations. Considering this dynamic nature of prefrontal attentional information results in variations by up to 10% of decoding accuracy and enhances the predictive power of whether the subjects will correctly respond to a target, miss the target or else miss-respond to a distractor.

### What cross-temporal decoding tells us about the brain

Cognitive processes in the brain are non-stationary and they evolve in time. In this context, neural decoding has shown to be a powerful tool to describe the temporal dynamics of coding information, describing the evolution of information decoding performance in time. For example, Bae (2020) studied the different time course differences between face identification (recognition) and face expression (emotion) processes using EEG recordings. In this study, participants performed a working memory task in which they were asked to remember a face image presented in the screen and, after a short delay, they were asked to report either the identity or the expression of the face. Importantly, participants had no *a priori* knowledge about the information they would be asked to report, thus, they had to extract and maintain both types of information during each trial using whole scalp raw data. In order to decode these two types of information, Bae and Luck (2018) used a combination of support vector machine and error-correcting output coded (Dietterich and Bakiri, 1994) to classify either facial expression of facial identification by using the scalp distribution of the phase-locked ERP voltage in the alpha-band activity (8–12 Hz). The decoding performance of both types of information was tracked in time, showing that these types of information exhibited a dissociated temporal dynamic. More specifically, decoding of the identification of the face was more prominent only during the time interval corresponding to the perception of the image, while the decoding of the face expression was more prominent during working memory maintenance, thus, along the whole trial duration. This result suggests that the neural representation of face identity and face expressions were, at least partially, independent. In addition, Bae and Luck (2018) uses the decoding performance as a tool to understand the temporal dynamics of encoded information not only within the same trial, but also between trials. Interestingly, they succeeded in decoding the identification of the face in the current trial using the information encoded from the previous trial. This did not apply to the decoding of face expression information. Therefore, this result suggested that neural decoding might be a useful tool to study how information encoded in the past can be reactivated regardless of its relevance for the current goal of the task.

Other studies have used neural decoding to study the dynamics of the hippocampal replay. Davidson et al. (2009) recorded multiple single unit activity in the hippocampal area CA1 in rodents while those were exploring a track. Using a probabilistic neural decoding strategy to estimate the animal’s position on the track from the spike trains, they evaluated whether recorded cells replayed spatial memory sequences (Brown et al., 1998; Zhang et al., 1998). Therefore, they conceived a neural decoding approach specific for replay detection. Interestingly, when rodents stopped exploring the environment, they showed signatures of time-compressed forward and reverse hippocampal replay of long behavioral sequences that, in turn, were associated with trains of ripple events. In addition, they found that replay was neither limited to locations associated with the reward, nor to those locations tied to the animal’s current location. Other studies have shown evidence of replay using non-invasive brain recordings in humans. Kurth-Nelson et al. (2016) recorded MEG activity from a cohort of human subjects while they were performing a non-spatial reasoning task. The aim of this study consisted in finding sequences of neural representations associated with learning and online planning, similarly to those found by replay, but in a non-spatial context. Indeed, the task required selecting paths through a set of six visual objects. They trained pattern classifiers on the MEG activity evoked by the direct presentation of the objects alone. Posteriorly, they tested these classifiers using the activity obtained during periods when no object was presented. They show that brain activity encodes the representation of at least four objects that were presented sequentially, following backward trajectories along the paths in the task. This was one of the first studies showing clear signatures of replay using non-invasive methods of brain recordings.

Decoding can also be used to investigate brain networks dynamics and coding regime. In this context, several studies have used cross-temporal decoding (King and Dehaene, 2014; Astrand et al., 2015; Amengual and Ben Hamed, 2021), which consists in training models at a given time in the trial and testing these models all throughout the trial time. This method allows discriminating different computational properties of the neural population, between stable coding regime (whereby the code identified at a given time generalizes at all times, indicating a stable coding schema by the underlying neuronal population, Figure 2C), and dynamic computational/cognitive processes (whereby the neuronal code identified at a given time does not generalize at other times, indicating a recurrent dynamic coding schema by the underlying neuronal population; Figure 2C). Astrand et al. (2015) studied the different dynamics of population coding during a spatial attention task using parietal and prefrontal electrophysiology recordings in macaques. Cross-temporal decoding matrices were used to determine whether tow brain regions, the frontal eye field (FEF) and the lateral intraparietal area (LIP) were presenting stable or dynamic coding. They show that the spatial attention code identified in the FEF at any given time can generalize to other times, thus indicating a stable coding schema. Indeed, this coding regime characterizes regions with activation dynamics mimicking those observed in artificial recurrent neural networks (Buonomano and Maass, 2009). Conversely, population activity in LIP showed a dynamic coding regime when decoding spatial attention, variable from one time to the next within the same trial. Therefore, these results show that neural decoding is a very suitable tool to study how different neural populations encode the same type of information by using different coding regimes.

In this line, other studies have addressed the question on how decision making might exploit cognitive flexibility to adapt behavior. Using time-resolved population-level neural pattern analysis from intracranial recordings from the prefrontal cortex in macaques, Stokes et al. (2013) investigated how context is encoded and maintained in the neural population in order to be exploited behaviorally. Specifically, they show that an instruction cue indicating the context of the trial in the task triggers a dynamic coding at trial onset, while the same information during the delay period prior to the decision is coded by a stable low energy state (Stokes et al., 2013). This method allowed the extraction of hidden patterns of activity in the data characterized by high and low energy states that tuned the prefrontal cortex according with the task demands. This observation extends to human brain function. This tuning mechanisms by hidden population states has been also explored in humans using non-invasive electrophysiology. In a series of studies, Wolff et al. (2015, 2017) ask participants to perform a working memory task while they record concomitant brain activity using EEG and MEG. In some of the trials, they presented a non-informative image (called impulse) during the period of working memory maintenance. They trained a decoder to predict the memorized item using activity previous to the impulse presentation (pure memory activity), or on the activity elicited by the task-irrelevant probe The authors show that the impulse generates a dynamic coding of the memory item which does not generalize to the other testing times. Taken together, these studies support the theory of hidden coding low energy states, whereby working memory information is encoded dynamically and reactivated by task-relevant but also by task irrelevant items (Wolff et al., 2015, 2017).

### What confusion matrices tell us about the brain

**FIGURE 3:**
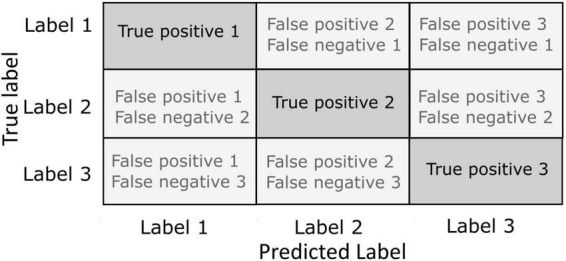
Confusion matrices. The instances of correct classification (true positives) and misclassifications (false negatives or false positives) are represented in a matrix with each row representing the actual true labels to be classified and each column representing the predicted label by the outcome of the classifier. Incorrect classification can be defined against the actual true label as a false negative or against the predicted label as a false positive.

Until know, we have discussed how decoding performance and their variation in time provides unique information about the cognitive processes underlying behavior. The question we will tackle in this section is whether we can extract genuine knowledge about cognition by studying how decoding algorithms fail in classifying information on which they have been trained. Indeed, studying how a decoder fails may also be very informative about cognitive brain processes. In this context, confusion matrixes are used to quantify the miss-classifications produced by a given decoder (Kriegeskorte and Kreiman, 2011). Confusion matrices allow a fine-grained analysis of the performance of the decoding algorithms in terms of hit rates and errors. Specifically, it permits to visualize the number of correct and incorrect prediction for each label (Figure 3). In a confusion matrix, each row represents one of the labels to be predicted, and each column represents the actual output of the classifier. In other words, for any given label presented along the rows, the confusion matrix presents the count of classification outputs assigned to that specific label but also to each of the other labels. A label correctly classified is considered as a true positive classification. When a label is not correctly classified, this is considered as a false negative for the targeted label, and as a false negative for the predicted label. For example, the study of confusion matrices when decoding covert attention using fMRI shows clear attentional biases toward the lower visual field or along the horizontal and vertical meridians such that decoding accuracy is up to 10% higher at these locations relative to other locations in the visual field, thus confirming attentional biased observed behaviorally (Zenon et al., 2008; Zénon et al., 2009; Loriette et al., 2021). In another fMRI study, Kim et al. (2019) studied how the brain visually encodes tactile intensities. To do this, they used an associative learning method to decode the representation of tactile intensities (roughness) evoked either by tactile exploration only, or by visual observation of the tactile exploration. In this work, they show that the behavioral data obtained while evaluating roughness during tactile exploration or visuo-tactile exploration correlates with the confusion matrices obtained when they decoded the roughness based on fMRI brain activity. In particular, this correlation was specific of the supramarginal gyrus, suggesting its role in tactile discrimination (Kim et al., 2019). Confusion matrices have also provided insightful information for the development of categorical models of emotions. Saarimäki et al. (2016) acquired fMRI data while participants participated in a task consisting in the identification of different fine emotions such as “joyful,” “amazed,” or “nervous,” embedded into basic emotion groups like “happiness,” “surprise,” or” fear.” Using multivariate pattern analysis (MVPA), they classified emotions from the activation in different brain areas including medial and inferior lateral prefrontal cortices, frontal pole, precentral and postcentral gyri, precuneus and posterior cingulate cortex. Importantly, participants behaviorally tended to misclassify emotions which are in the same group and these misclassifications were similar to those obtained by decoding emotions from fMRI signal, suggesting a link between the activity in these regions and the emotional perception (Saarimäki et al., 2016). Electrophysiological studies have been benefited as well from the use of confusion matrices to evaluate the decoding capacities of the brain signal. Chen et al. (2019) developed a LFP-based close-loop deep brain stimulation strategy in Parkinson patients implanted with electrodes in the subthalamic nucleus. The aim of this method was to adapt the stimulation interval based on the sleep stage (Amara et al., 2016). Therefore, machine learning methods were applied to decode in real time the sleep stage of the patient from the LFP recorded from the DBS leads. Based on different combinations of signal features extracted within the time domain, frequency domain and variance of the signal, they succeeded in decode the sleep stage from the LFP in this region using a SVM-based algorithm. However, confusion matrix revealed that not all sleep stages were decoded with the same accuracy: while the decoder was able to accurately classify wakefulness against sleep and N2 phase against REM, phase N1 and REM showed a high degree of confusion, although still above chance level. Therefore, this study showed that subthalamic nucleus LFP-based activity encode information about the brain state activity (but only some of these states). Other studies have addressed the question of the regional- and modality-specificity of the decoding capacities of the cortex. Enander et al. (2019) delivered a set of electrical spatiotemporal tactile afferent activation patterns to the skin of the contralateral second digit of the forepaw in anesthetized rats. Concomitantly to this stimulation, there performed *in vivo* single neuron recordings in the right hemisphere, more specifically in the primary somatosensory cortex, but also in other cortical locations within and out of the somatosensory cortex. They used the recorded brain activity of these neurons to decode the different stimulation patterns delivered by the electrical stimulation. Interestingly, they found that neurons from the primary sensorimotor cortex showed similar decoding performance compared with neurons from out of this area. Indeed, confusion matrix revealed that neurons from regions of the visual cortex showed less miss-classification rates than those in the sensorimotor cortex. This study provided direct evidence on how the tactile information could be propagated globally across the neocortex, presumably *via* cortico-cortical but also cortico-thalamo-cortical pathways (Lübke and Feldmeyer, 2007; Frostig et al., 2008).

### Exploring shared functional substrates amongst different cognitive tasks

Another possible application of decoding methods is to evaluate the decoding performance of a decoder trained to decode a specific type of information (e.g., position) of a cognitive process (e.g., memory) and testing this neural decoding on a different information (e.g., color) or cognitive modality (e.g., attention). By using this method, it is possible to identify informational communalities between both sources of information or between two cognitive processes. We selected two different fMRI studies that exemplify this point (Albers et al., 2013; Dijkstra et al., 2019). In these studies, participants were presented either with a visual stimulus, or asked to mentally imagine these stimuli. Interestingly, both studies succeeded in decoding each of the two cognitive processes (mental imagery and visual perception) by using a decoder trained on either mental imagery brain activities or visual perception related brain activities, indicating that mental imagery coding and visual perception share similar cortical representation.

### Exploring model parameters to model the brain

Until now, we have shown that neural decoding accuracy provides information about a broad series of cognitive processes and their neural underpinnings. However, other elements associated with the neural decoding algorithms, such as the parameters obtained from the decoding model (e.g., the weights that are fit in linear regression), can be exploited to study cognitive brain functions. One of these parameters is the number of extracted features. This number is obtained by a process consisting in extracting a small number of features that maximizes the information about the statistical structure of the data. Numerous methods are available in order to perform feature selection, ranging from statistical test as ANOVA, to principal component analysis (PCA), mutual information maximization, searchlight and others (Pedregosa et al., 2011; Abraham et al., 2014; Allefeld and Haynes, 2014; Cunningham and Yu, 2014; Padmanaban et al., 2018). For example, in fMRI, an ANOVA-based feature selection precisely reveals the cortical topography for covert visual attention to guide single trial fMRI-based spatial decoding of attention (Loriette et al., 2021). Another type of feature selection, called searchlight, aims at searching for the most informative features and select them to train the decoder, by looking at how each feature separately contributes to improving the classification accuracy. Stokes et al. (2009) investigated mental imagery in an fMRI study with human participants. Authors compared the classification accuracy in different fMRI regions while participants imagined different letters. This method allowed to identify high informative areas in mental imagery [including inferior occipital gyrus (IOG), middle occipital gyrus (MOG), fusiform gyrus (FG), middle temporal gyrus (MTG) and temporal gyrus/Heschl’s gyrus (STG/HG)]. When comparing these areas to the ones obtained from a searchlight procedure on brain activity elicited by visual stimuli, the authors further identify cortical regions that are involved in mental imagery and not in pure visual perception (STG/HG) (Stokes et al., 2009). Another study compared searchlight method to 3D classical fMRI analysis, showing that the searchlight method is more spatially specific compared to classical methods (Chen et al., 2011). This result indicates that this method outperforms classical statistical tests and reveal very precise local functional selectivity patterns in brain areas that were initially thought as functionally homogenous. Other studies have succeeded in using EEG recordings to decode semantic information. One of the relevant questions here is what kind of signal (or pattern) might provide more exploitable semantic information to be used for decoding. For instance, Jafakesh et al. (2016) used different features of the recorded EEG signal to decode the semantic category of different visual stimuli. Specifically, they used several within electrode cross-frequency coupling (CFC) measures such as amplitude-amplitude coupling (AAC), phase-amplitude coupling (PAC), and phase-phase coupling (PPC) within each electrode and used them as input to SVM classifier. They found a higher decoding performance using PPC than using the other two measures, specifically in the alpha and gamma frequency bands. In addition, they tested whether using CFC-based measures to classify semantic information outperformed the decoding with respect of using wavelet transform of the EEG signal. In this context, they obtained a higher decoding performance using PAC relative to using wavelet coefficient. Therefore, CFC measures provide information regarding semantics that is not available in the time-frequency components.

Although there is not a clear consensus on their interpretation, the weights of the decoder are often used to analyze informative cortical voxels, under some conditions. Generally speaking, weights represent the accountability of the information content of each feature which is fed into the decoding algorithm (Kriegeskorte and Bandettini, 2007; Haufe et al., 2014; Kia et al., 2017; Hebart and Baker, 2018). As an example, weights extracted from Linear Discriminant Analysis (LDA) model in EEG while performing gesture classification at the single trial level reveals the frequency and the channels which are the most informative during walk preparation (Velu and de Sa, 2013). In contrast, weights extracted using a linear regression analysis on intra-cortical neuronal data to decode spatial attention from the single trial activity of a prefrontal or a parietal neuronal population can only be interpreted when normalized by the average response of each neuron (Astrand et al., 2015). As a result, high weights associated with weak average neuronal responses might turn out less informative than smaller weights associated with high average neuronal responses. In other types of decoders yet, weights cannot be readily interpreted (Haufe et al., 2014; Kriegeskorte and Douglas, 2019).

## Interfering with brain activity with neurofeedback or learning

### Brain–machine interfaces

**FIGURE 4:**
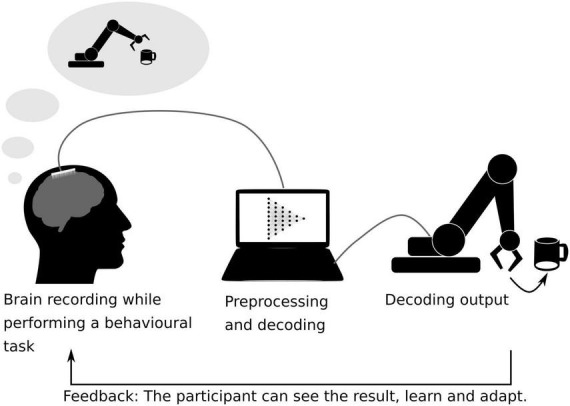
Schematic representation of a closed-loop brain–machine interface. The brain activity is recorded while the subject is performing a behavioral task. Here, the participant performs a motor-task and tries to grab a coffee cup with a robotic arm, while her/his brain activity is being recorded using ECoG sensors. The recorded data is pre-processed and fed into a machine learning algorithm previously trained to interpret the participant’s motor intentions (Hochberg et al., 2012). The decoded movement is translated into a robotic arm movement. The actual movement of the robotic arm serves as a feedback for the participant on her/his brain activity, allowing her/him to learn control the device.

Although the scope of this review is to present the neural decoding as a tool not only used to develop strategies for neuroprosthetics but also to understand cognitive function, we found it necessary to discuss some examples on how decoding of cognitive information has been used to develop brain–computer interfaces (BCI) in turn producing knowledge on brain organization and plasticity. BCIs are direct or indirect communication interfaces between the brain and a computer. These methods rely on closed-loop systems, which refers to the fact of providing to the subject a direct (e.g., MUA or BOLD activation level in a specific cortical region) or an indirect (e.g., decoded information across a set of MUA, EEG or BOLD signals, or signal coherence across multiple EEG channels) feedback extracted from brain activity (Chaudhary et al., 2016). One example of BCIs is the neuroprosthetic BCIs, aiming at replacing a deficient brain function. For example, motor neuroprosthetics have been developed to allow tetraplegic patients to control a robotic arm thanks to the real-time decoding of ECoG recordings (Hochberg et al., 2012; Bensmaia and Miller, 2014). Sensory neuroprostheses have also been developed to restore tactile sensation, for example injecting complex microstimulation patterns into the somatosensory cortex of macaque monkeys to generate artificial sensations guided by touch sensors implemented in a robot arm (O’Doherty et al., 2011) (Figure 4).

These decoding approaches allow not only to explore brain functions but also to train them, by providing a feedback to the participant or the animal about their brain activity (raw or processed/decoded) for them to act on it to improve behavior. This closed-loop procedure is called neurofeedback (Sitaram et al., 2017). One example of these neurofeedback tools is the P300 speller, which is an EEG-based BCI. This method consists in spelling a word that the participant has in mind by attending to a target letter presented in the midst of other letters (all alphabet letters being covered sequentially) by flashing a sequence of letters alternating between rows and columns. When the letter selected by the participant is flashed, the evoked response in the brain is stronger and the decoder can exploit this difference to identify the selected letter amongst the presented letters (Guy et al., 2018). Performing with the P300 speller trains subjects to flexibly use their attentional resources and this results in an enhancement of their attentional performance beyond this specific task (Arvaneh et al., 2019). Neurofeedback has also been implemented in fMRI protocols on categorical attention in order to increase behavior as well as attention-related brain information as assessed by the decoder’s accuracy (deBettencourt et al., 2015).

Importantly, neurofeedback has additionally been used in order to investigate the effects of interfering with brain functions. For example, an fMRI study shows that it is possible to selectively increase or decrease a participant’s confidence in her/his performance during a behavioral task without interfering in her/his actual success rate in the task using neurofeedback specifically based on the decoded participant’s confidence (Cortese et al., 2016). In another fMRI study, participants were able to learn to associate red or green color with different grating orientations. Specifically, after training a decoder to discriminate the color the participants were presented with (green or red), participants were trained to modulate their brain activity while a grating with a specific orientation was displayed. If the grating was vertical, participants had to modulate their brain activity in order to increase the likelihood of decoding red color in the visual cortex. If the grating was horizontal, participants were asked to increase the likelihood of decoding “green.” As a result, after neurofeedback training, when presented with vertical or horizontal achromatic gratings, the participants tended to perceive them in the color they were trained to modulate their activity in association with the grating orientation. Surprisingly, these effects persisted up to 5 months (Amano et al., 2016).

There is an ever-growing number of neurofeedback studies and tools aiming at both training cognitive functions and understanding brain functions. In particular, this approach has been shown to potentiate brain plasticity, triggering not only behavioral long term effects, but also plastic changes in brain structure and anatomy, such as an increase in gray matter volume and white matter myelinization (Ghaziri et al., 2013; Marins et al., 2019; Loriette and Ziane, 2021).

An important field of application of brain decoding and neurofeedback relies on using brain–machine interfaces (BCIs) in order to restore an acute or chronic brain deficits for neurorehabilitation (Chaudhary et al., 2016; Lebedev and Nicolelis, 2017; Bockbrader et al., 2018). Impressive advances have been achieved in BCI-driven motor rehabilitation after stroke (Wang et al., 2018) or spinal cord injury (Pohlmeyer et al., 2009; Bensmaia and Miller, 2014; Ajiboye et al., 2017). As an example, Pohlmeyer et al. (2009) used a cortical controlled electrical stimulation of the forelimb of spinal cord injured macaques and managed to restore voluntary movement. Another study using EEG BCI coupled with electrical stimulation restored hand movements in patients after a stroke injury, this restoration persisting 6–12 months after the BCI training (Biasiucci et al., 2018).

### Manifold coding and learning, a new approach to understand cognition

**FIGURE 5:**
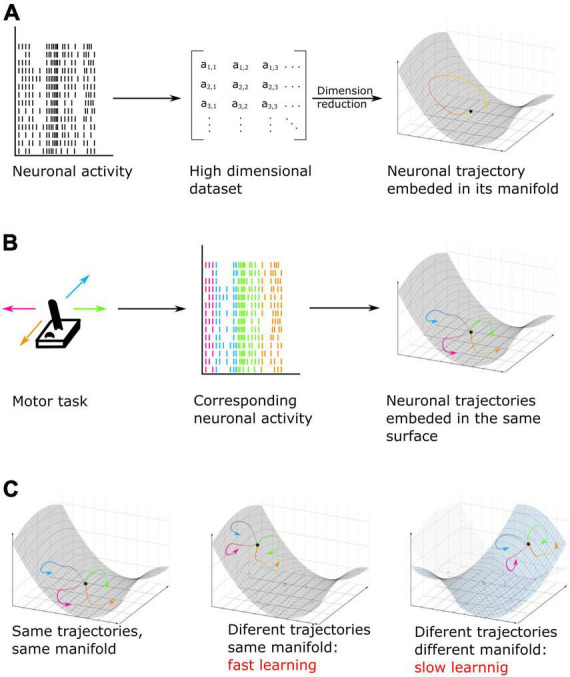
**(A)** Neuronal activity, here MUA recordings, taken as an example, is converted into a lower dimensional space called manifold. In this low dimensional surface, the neural code can be represented as data points or trajectories when the pattern of activity is analyzed over successive time epochs. **(B)** Example of a motor task. Here, neuronal activity is recorded while subjects move a joystick in one of four possible directions. On individual trials, each movement can be represented by a different pattern of activity or trajectory but all trajectories are embedded in the same neuronal low dimensional manifold neural space [based on Gallego et al. (2017, 2018)]. **(C)** Example of cognitive training using manifold perturbation. Participants or animals are given a feedback (for example, cursor velocity) and have to learn to control it by using the same neuronal pattern as recorded during previous tasks (left) (for example, during a motor task). It is also possible to train subjects by using a different pattern embed in the original manifold (center) or in a new manifold surface (right). The last option leads to a slower learning and an increased difficulty of the task (based on Sadtler et al., 2014; Golub et al., 2018; Oby et al., 2019).

Recording brain activity with the most up-to-date recording methods result in very high dimensional data sets. This can be a limiting factor in decoding speed. Techniques such as principal component analysis (PCA) (Cunningham and Yu, 2014), independent component analysis (ICA) (Brown et al., 2001) and their derivatives have been developed to reduce the dimension of the dataset. Importantly, such dimensionality reduction methods, in addition to compressing dataset size provide a very unique insight in how the neural code is encoded in the brain. These low-dimensional spaces which allow to analyze patterns of activity and interaction between neurons are called neural manifold (Elsayed and Cunningham, 2017; Degenhart et al., 2020). These manifolds can be seen as low dimensional spaces that reflect neural modes (Figure 5A), that is to say, patterns of activity corresponding to cognitive actions which can be embedded into a surface. The response activity from one cognitive task (for example moving the arm) can be tuned differently depending on little variations of this task but the activity remains embedded in the same surface, while a second action (for example walking around) will be embedded in a different manifold. As an example, moving an arm in a certain direction can be seen as a trajectory in a three-dimensional surface. Each movement direction is embedded in this surface but can be separated visually and mathematically one from the other (Gallego et al., 2017, 2018) (Figure 5B). While dramatically enhancing our understanding of how the brain copes with the dimensionality of the information, decisions and cognitive functions it has to implement (Rigotti et al., 2013), it also allows to enhance brain computer interfaces techniques, accelerating computational speed as well as allowing to cope with neural instability by realigning recorded activity to its original manifold before performing decoding, thus maintaining constant decoding accuracy in time (Degenhart et al., 2020; Gallego et al., 2020).

The theory of neural patterns embedded into manifolds has been specifically applied in order to investigate learning, which by definition impacts neuronal computation as learning progresses. For example, researchers have used brain computer interfaces in order to train monkeys to control the velocity of a cursor by using the neuronal activity generated by their motor cortex. They first decoded arm velocity from the neuronal activity of the arm region of the primary motor cortex. Then, they trained the monkey to control the velocity of the cursor, not based on the pre-existing code assessed in the first step, but either using a new activity pattern embedded into the original manifold, or using a new activity pattern positioned outside of the original manifold (Figure 5C). They found that monkeys learn faster if the new pattern of activity they have to learn is embedded in the original manifold (Sadtler et al., 2014; Golub et al., 2018). Moreover, researchers found that forcing the monkey to learn a new neural code out of the original manifold is not only slower, but leads to the emergence of a new neural mode, in other words, a new manifold in which the neural code will be embedded (Oby et al., 2019). Overall, these studies result in a new understanding of how learning is implemented in the brain and how it can best be potentiated. This substantiates why learning complex and new tasks is slower than learning familiar tasks, as this requires creating new patterns of activity that do not interfere with prior learning (Figure 5C).

## Deep learning: A window onto the complexity of brain functions

The vast majority of decoding algorithms used in the examples cited in this review are based on learning certain rules of inference that are used to estimate linear or non-linear prediction functions to map input activity onto a set of outputs. Importantly, these algorithms do not learn neural representations directly, but are trained to determine decision boundaries that are used to classify the inputs onto specific outputs (Amengual and Ben Hamed, 2021). However, some non-linear properties of these representation and their large dimensionality prevent the optimal performance of these classification algorithms and, thus, additional preprocessing methods based on reducing data dimensionality and feature extraction techniques are needed (Blum and Langley, 1997; Schölkopf et al., 2007; Abrol et al., 2021). The application of these preprocessing steps on input data imposes certain *a priori* assumptions and requires a certain high expertise of the users, which reduces part of the automatization of the knowledge extraction process. As a response to this need, deep learning approaches have been introduced for neural decoding purposes. Differently to standard machine learning approaches, deep learning characterizes patterns embedded in the raw data as a part of the training process. To do this, deep learning models are based on multiple layers artificial neural networks (ANN) that allow to progressively extract high-level features from input data. These models consist in a composition of components that are formed by linear and non-linear operations forming complex layered architectures. Some examples of these networks are the recurrent neural networks (RNN), which are suitable to model the temporal dynamic behavior of a given process, convolutional neural networks (CNN), most commonly applied to analyze visual imagery and long short-term memory (LSTM) networks, a particular case of RNN with feedback connections, suitable to model memory processes. In the following we will discuss some applications of the deep learning methods for neural decoding in vision.

Indeed, decoding visual stimuli, understood as the capacity to predict the identity (meaning) or physical attributes of visual stimuli by using brain activity, represents a major challenge in neuroscience. Seminal fMRI studies have shown that visual features such as orientation, motion direction and visual object categories can be decoded from BOLD signal recorded over the visual cortex and ventral parietal cortex (Haxby et al., 2001; Cox and Savoy, 2003; Kamitani and Tong, 2005, 2006). However, these studies used the activation from voxels in selected visual cortices to feed into the decoding algorithm and, thus, they did not take into account the internal relationship between the different visual areas. Indeed, it is well known that a higher level function such as object recognition requires the co-activation of different brain areas in a hierarchical manner along the ventral stream (Mishkin et al., 1983). Anatomical studies have found that connections between the different layers of the ventral stream are bidirectional (Bar, 2003). These forward and backward connections provide the anatomical substrate of the information flow in the visual cortex. In this context, visual information might flow from primary visual cortices toward high-level visual cortices to obtain high-level semantic understanding [bottom-up visual mechanisms, (Logothetis and Sheinberg, 1996)]. In turn, visual information feedbacks from high-level to low-level visual cortices, which is known the top-down visual mechanisms (McMains and Kastner, 2011). In order to succeed in neural decoding of object recognition, Qiao et al. (2019) conceived the use of a RNN in which they split the neurons into positive and negative directions and fed the activity of each voxel in each visual area while participants observed natural photographs. In this way, they did not model only the information of each visual area, but also the internal relationship between the different visual cortices, in the decoding method. Comparing the decoding accuracy of this method with the accuracy obtained using other classical classifiers such as decision trees and random forest, they found that this method of decoding improved the classical decoding methods by a 5% of accuracy, on average. In addition, they concluded that the representation in visual cortices were hierarchical, distributed and complementary, since the increment in decoding performance depended on the conception of the multiple layers simultaneously. Other studies have tried to use deep learning approaches to decode brain’s responses to representation of natural video stimuli. To this end, Wen et al. (2018) acquired very long fMRI acquisitions of three human subjects watching 972 different video clips that included diverse scenes and actions. The aim of this study was to use a convolutional neural network (CNN) in order to reconstruct and categorize the visual stimuli based on the fMRI activity recorded from the dorsal and ventral streams in a dynamic condition. They found that the CNN was able to predict non-linear and complex patterns of responses in both dorsal and ventral streams, with a high decoding accuracy in category representation. Indeed, the CNN supported the reconstruction of decoded natural movies and direct semantic categorization. All in all, these studies exemplify how deep learning algorithms can decode visual information with a very high degree of specificity. It is expected that such methods can be generalized in the future to the read out of more complex cognitive functions.

## Conclusion

Advances in brain activity recording and processing and in machine learning have led, in these recent years, to a new way of exploring brain function. In fact, it is now possible to access to a better understanding of brain function using recorded activity, as for example while trying to infer the spatial location of covert attention in real-time in macaques or humans. Decoding neuronal brain activity can be performed with both invasive and non-invasive techniques, each presenting its pros and cons. It permits to have a direct access to a part of brain information and build brain–machine interfaces as for example, controlling a robotic arm with motor cortex activity. But these major advances performed in this field in these last years did not only permit to perform “mind reading” of the brain. These methods have also generated robust statistical tools to better understand brain function and cognition. In this review, we have explored brain decoding approaches, not only from the perspective of inferring hidden brain states but also from the perspective of understanding brain functions. For example, exploring decoding accuracy allows to explore the temporality and the stability of brain processes (Astrand et al., 2015; Wolff et al., 2015, 2017) while searchlight methods or decoder weights analysis allows to extract a refined view of how the brain organizes information processing at a high spatial resolution (Chen et al., 2011; Haufe et al., 2014). The development of these exploratory methods has resulted in new hypotheses about how neural networks can encode a given function and even how this code can be modified by learning. As an example, numerous articles are now exploring the theory of manifolds embedding neural activity (Gallego et al., 2017, 2018).

These advances in decoding and computational neurosciences open the way of combining different brain activity modalities when exploring any given function (e.g., EEG and fMRI or MUA and LFP). Indeed, each recording technique can bring specific information. This is expected to enhance our understanding of brain functions and allow to explore differences in information content between data collected simultaneously in different modalities. One thinks of differences in temporal and spatial resolution, but other functional differences are also increasingly reported, as for example in the informational content of spikes versus LFP (Pesaran et al., 2002; Perel et al., 2013).

The road is still long before a full exploitation of all of the potentialities of this new research field which combines a mechanistic understanding of the brain with machine learning tools (Pedregosa et al., 2011; Savage, 2019; Glaser et al., 2020; Iturrate et al., 2020). Brain computer interfaces and neurofeedback protocols are still at their early days and will probably benefit from the continuous progresses observed in computational sciences.

## Author contributions

CL and SBH: conception of the review. CL, SBH, and JA: write draft and corrections. All authors contributed to the article and approved the submitted version.
